# Management of recurrence after peroral endoscopic myotomy and submucosal tunneling endoscopic septum division

**DOI:** 10.1055/a-2336-6554

**Published:** 2024-06-25

**Authors:** Ke-Yang Fan, Meng-Jiang He, Li Wang, Jia-Qi Xu, Quan-Lin Li, Ping-Hong Zhou

**Affiliations:** 1 Endoscopy Center and Endoscopy Research Institute, Zhongshan Hospital, Fudan University, Shanghai, China; 2 Shanghai Collaborative Innovation Center of Endoscopy, Shanghai, China

Achalasia sometimes coexists with esophageal diverticulum 1 , and the combination of peroral endoscopic myotomy (POEM) and submucosal tunneling endoscopic septum division (STESD) is efficient and safe for relieving the symptom 2 3 . However, the management of symptom recurrence after this combination of procedures is more challenging due to fibrosis of the submucosa. We present a case of short-term recurrence in a teenager after POEM and STESD, in which achalasia and epiphrenic diverticulum were treated through a repeat POEM procedure.

A 13-year-old girl was admitted to a local hospital with achalasia and mid-esophageal diverticulum, and underwent POEM and STESD ( Fig. 1 **a–e** ). The symptoms recurred 3 months after the surgery and responded poorly to balloon dilation. The patient visited our hospital 6 months after the first surgery. Barium esophagography indicated barium retention, and a newly developed epiphrenic diverticulum ( Fig. 1 **f** ). As this short-term recurrence was caused by incomplete myotomy rather than scar formation or disease progression, the multidisciplinary team scheduled a repeat POEM procedure to alleviate the high pressure, while also treating the epiphrenic diverticulum without septum division ( Video 1 ).

**Fig. 1:**
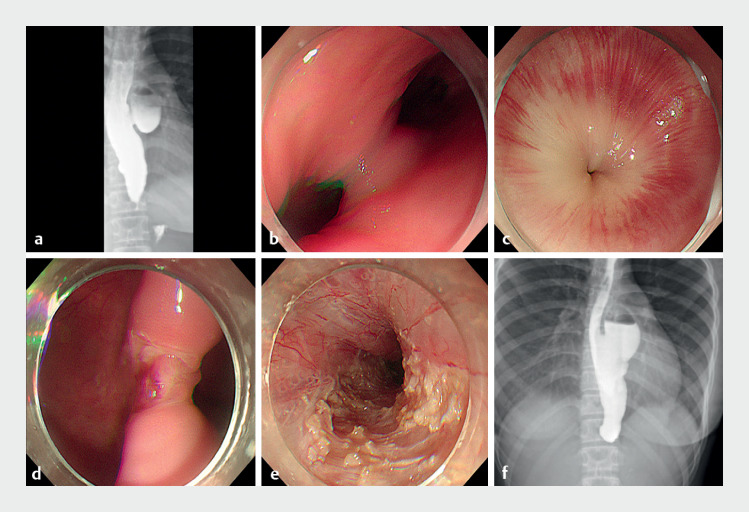
Initial diagnostic and therapeutic history. **a** Preoperative esophagography. **b** Endoscopic view of the mid-esophageal diverticulum. **c** Endoscopic view of the tight cardia. **d, e** Surgical images of submucosal tunneling endoscopic septum division and peroral endoscopic myotomy. **f** Postoperative esophagography.

Repeat peroral endoscopic myotomy (POEM) for recurrence of symptoms after POEM and submucosal tunneling endoscopic septum division.Video 1

After rotating the endoscope clockwise by 180 degrees, we created the mucosal entry on the opposite side to the previous entry to avoid the fibrotic submucosa, and extended the submucosal tunnel to 3 cm below the cardia ( Fig. 2 **a, b** ). Then, we dissected the circular muscle, and performed total myotomy 2 cm above and below the cardia ( Fig. 2 **c** ). The cardia was noticeably enlarged after the myotomy ( Fig. 2 **d** ). The entry was closed after hemostasis ( Fig. 2 **e** ).

**Fig. 2:**
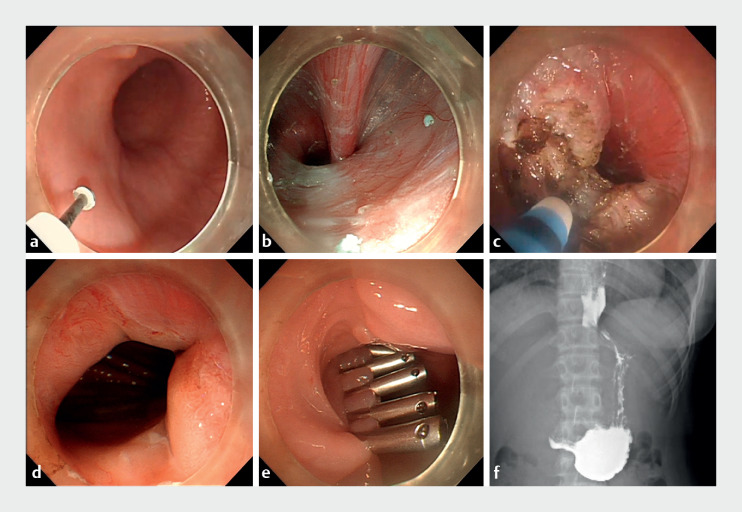
Repeat peroral endoscopic myotomy and follow-up. **a** Mucosal entry creation. **b** Submucosal tunnel creation. **c** Total myotomy at the cardia. **d** Open cardia. **e** Closure of the entry. **f** Postoperative esophagography.

At the 3-month follow-up, the patient reported a weight gain of 3 kg, and there was limited barium retention in the esophagus ( Fig. 2 **f** ).

Although epiphrenic diverticula are typically caused by long-term high pressure, they can also occur as a short-term complication after POEM. For epiphrenic diverticula without obvious septum caused by pressure, additional STESD is not necessary 4 .

Endoscopy_UCTN_Code_TTT_1AO_2AP

## Notes

Endoscopy E-Videos https://eref.thieme.de/e-videos
*E-Videos* is an open access online section of the journal *Endoscopy* , reporting on interesting cases and new techniques in gastroenterological endoscopy. All papers include a high-quality video and are published with a Creative Commons CC-BY license. Endoscopy E-Videos qualify for HINARI discounts and waivers and eligibility is automatically checked during the submission process. We grant 100% waivers to articles whose corresponding authors are based in Group A countries and 50% waivers to those who are based in Group B countries as classified by Research4Life (see: https://www.research4life.org/access/eligibility/ ). This section has its own submission website at https://mc.manuscriptcentral.com/e-videos .
